# We are MLA: a qualitative case study on the Medical Library Association's 2019 Communities Transition

**DOI:** 10.5195/jmla.2022.1225

**Published:** 2022-01-01

**Authors:** Kathryn M. Houk, Kelsa Bartley, Jane Morgan-Daniel, Elaina Vitale

**Affiliations:** 1 kathryn.houk@unlv.edu, Health Literacy & Community Engagement Librarian, Assistant Professor, UNLV Health Sciences Library, University of Nevada, Las Vegas, Las Vegas, NV; 2 k.bartley@med.miami.edu, Education and Outreach Librarian, Librarian Assistant Professor, Louis Calder Memorial Library, University of Miami Miller School of Medicine, Miami, FL; 3 morgandanie.jane@ufl.edu, Community Engagement & Health Literacy Librarian, Assistant University Librarian, Health Science Center Libraries, University of Florida, Gainesville, FL; 4 elaina.j.vitale@dartmouth.edu, Research and Education Librarian, Biomedical Libraries, Dartmouth College, Hanover, NH

**Keywords:** organizational change, change management, library association management, Medical Library Association, organizational communication

## Abstract

**Objective:**

In 2019, the Medical Library Association (MLA) adopted a new model of community governance and participation, referred to as the MLA Communities Transition. The Communities Transition was the culmination of long-ranging plans by MLA to support two of its strategic goals: diversity and inclusion, and communities. The reorganization aimed to strengthen MLA member communities, better support programming, reduce administrative overhead, and attract new members. The 2019–2020 MLA Rising Stars cohort was tasked to study the Communities Transition and identify lessons that might be applicable to any major future change proposed for the organization.

**Methods:**

A qualitative study was designed and conducted to investigate MLA member and leader perceptions of the change process, using John Kotter's eight steps for organizational change model as a framework. A set of fifteen open-ended questions was developed based on Kotter's model, and seventeen semistructured interviews were conducted to gather perceptions and feedback. Interview transcripts were analyzed using a grounded theory approach to explore and identify several themes across all discussions.

**Results:**

Four major themes were identified: communication between leadership and membership, leadership during the change process, membership investment in change, and instituting change and future recommendations. The study revealed strengths in the overall implementation and execution of the transition, but it also highlighted several perceived issues with communication and information sharing.

**Conclusions:**

Study findings were used to develop recommendations for improved communication strategies and for handling large-scale changes within the organization in the future.

## INTRODUCTION

The Medical Library Association (MLA) is the professional home for more than 3,000 librarians and information professionals working in health sciences, hospital, and medical settings. It is a member-driven association that aims to represent the voices of a diverse group of information professionals. MLA has provided professional development, education, and support to librarians for over one-hundred years, in exchange for annual membership dues. In 2019, MLA adopted a new model of community governance and participation, the MLA Communities Transition. Prior to the Transition, the organization had a two-level membership model, where members could pay additional yearly dues to belong to sections representing their work environments or fields of focus. For example, sections existed for hospital libraries, nursing and allied health librarians, and consumer health. Members could also join a variety of special interest groups (SIGs) at no cost.

**Figure 1:**
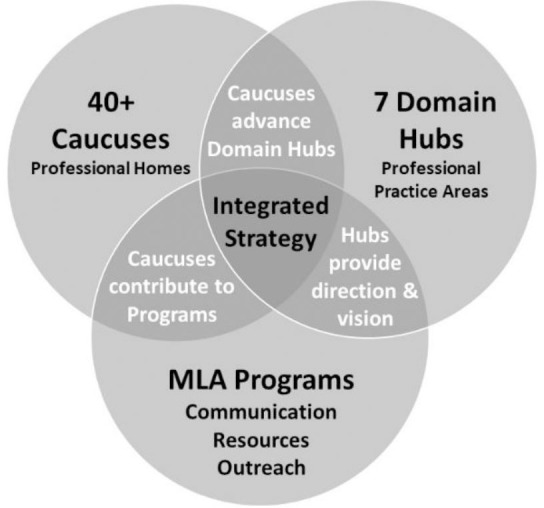
Collaboration, content, programming, and communication matrix. Reproduced with permission from MLA.

The Communities Transition initiative resulted from several years of behind-the-scenes work and was supported by two of MLA's strategic goals: 1) diversity and inclusion, and 2) communities [1]. Both executive and board leadership viewed the original two-level membership model as financially exclusive, because of the requirement to pay individual section dues on top of general membership dues, and aimed to create a more inclusive organization by reducing cost barriers and encouraging communication and collaboration across sections. MLA executive and board leadership also hoped to use the Transition to evaluate and improve MLA practices related to diversity and inclusion across MLA, including documents, programs, and publications [2]. In the final structure, SIGs and sections were transitioned into caucuses, none of which require membership dues. All caucuses are administered by a community council, and collaborative work is encouraged between caucuses through seven domain hubs aligned to areas of professional practice. Individual section treasuries were moved to MLA's central treasury to continue supporting scholarships. Overall, the Communities Transition aimed to strengthen MLA member communities, better support programs, reduce administrative overhead, and attract new members. Figure 1 illustrates the intersection of the caucuses, domain hubs, and MLA programs outreach under the new structure [2].

The Communities Transition was undertaken using John Kotter's eight-step model for organizational change as a guide. In his book, *Leading Change,* Kotter offers an eight-step model that guides readers from project inception to conclusion for organizations undergoing radical change. The model includes the steps:

- Create a sense of urgency
- Build a guiding coalition
- Form a strategic vision and initiatives
- Enlist a volunteer army
- Enable action by removing barriers
- Generate short-term wins
- Sustain acceleration
- Institute change [3]

This text informed MLA executive and board leadership strategies for the Communities Transition as well as the research and analysis described in the present study. As members of the 2019–2020 Rising Stars cohort, an MLA-sponsored leadership training program, we were tasked with investigating perceptions of the change process. Through a qualitative study using thematic analysis of a series of semistructured interviews, we developed quality improvement recommendations for MLA executive and board leadership, primarily around communication during organizational change.

## METHODS

A proposal for this qualitative study involving semistructured interviews with various MLA members in leadership positions was presented to the MLA Board of Directors in fall 2019 and approved. As this project was an internal study on practices and events related to MLA, it is considered a quality improvement study and not subject to Institutional Review Board oversight according to 45 CFR 46.102 Section I [4]. However, all participants were invited through an email designed to closely conform to ethical, informed consent standards (Appendix A). Interviewees were kept informed of study outcomes, and anonymized quotes are used with their explicit permission.

Fifteen open-ended questions were developed for this study (Appendix B) based on Kotter's eight-step model for organizational change [3]. Study participants were selected from MLA members who were involved in the Communities Transition process between 2015 and 2019. A list of twenty potential participants was generated by the authors and Rising Stars Program leaders under the direction that members in Communities Transition committees, section, or SIG leadership positions during the Communities Transition were to be prioritized. All study results should thus be interpreted with the understanding that there is likely bias due to underrepresentation of nonleadership input.

Eighteen members agreed to participate in our study, and interview questions were sent to them in advance of the video meetings. The final analysis included sixteen transcripts, as two individuals interviewed together and one interview lacked a recording transcript. Interviews took place via video conference from November 2019 through January 2020. At least two authors were present at each interview; one led the interview while the other took notes to aid in autogenerated transcript comprehension. Most interviews lasted between half an hour and one hour, as opportunities for discussion were provided following the question-and-answer sessions. Notes, video recordings, and transcripts were shared with all authors through privately available cloud storage. Prior to publication, interviewees were emailed for their consent to use their anonymized quotes, with sixteen consenting.

Transcripts were analyzed using a modification of the thematic analysis technique by Braun and Clarke [5]. Thematic analysis is a qualitative research approach that involves applying codes to written or transcribed documents, usually interviews, to uncover underlying themes across multiple documents. When working in a team, consensus coding occurs when team members agree on codes and their rules of application within the documents. In our approach, prior to reviewing documents, a list of consensus-based codes based on Kotter's model was created with the expectation that additional codes might be created by individual authors while reviewing transcripts. Each transcript was coded by two authors independently, preferably those who were not present during the original interview. Coded documents were then reviewed by a single author who synthesized codes into themes. In thematic analysis, themes are determined and influenced by the researchers' point of view and understanding of the topics in the data. All four authors are MLA members, went through the Rising Stars Program as a cohort, were not involved in Communities Transition leadership, and were aware of the Transition process. Accordingly, we had similar knowledge, biases, and experiences, allowing for agreement regarding interpretation of the interviews and the final synthesis and themes.

## RESULTS

Coding of the transcripts enabled the identification of repeated patterns or themes, through which a thematic map was created. In total, four themes were identified with sixteen subthemes, which closely mapped to the questions asked during the interviews. The results are presented narratively in accordance with the identified themes and subthemes (Table 1).

**Table 1:** Map of themes and subthemes identified during analysis.

Themes	Subthemes			
**Theme one: Perceived communication between leaders \& members**	Effectiveness of leadership communication strategies	Effective \& ineffective communication channels	Communication of need for change \& communities strategic goal	Understanding of need for change
**Theme two: Perceived leadership during change process**	Length of change process	Current pace of change \& overall pace of change process	Representativeness of transition leadership team	Power of the transition leadership team
**Theme three: Perceived membership investment in change**	Membership response to change: positive or negative	Reason for membership responses	Feedback solicitation by association leadership	Mechanisms used to solicit membership feedback
**Theme four: Instituting change \& future recommendations**	Clarifying \& communicating policies \& procedures to membership	Understanding short-\& long-term evaluation of success	Communicating successes \& challenges transparently	Consistently promoting new structure to encourage engagement

Theme one, Perceived communication between leaders and members, encompasses the perceived effectiveness of messaging from MLA leadership to the general membership, the choice of communication channels, and whether the information communicated effectively conveyed the reasons for the Communities Transition. Theme two, Perceived leadership during the change process, involves representation, power dynamics, and perceptions of the pace, process, and length of the Transition. Theme three, Perceived membership investment in change, incorporates the general membership's responses to the change, the potential reasons for these reactions, and leadership solicitation of member feedback. Theme four, Instituting change and future recommendations, arose from participant discussions on how best to solidify the Communities Table 1 Map of themes and subthemes identified during analysis. Transition and help MLA membership effectively engage with the association into the future. This theme encompasses interviewees' suggestions for clarifying and communicating the new organizational structure, policies, and procedures to membership, as well as evaluating the Transition's short- and long-term success.

**Theme one: Perceived communication between leaders and members**

### Effectiveness of leadership communication strategies

The interview question “Did MLA communicate effectively with the membership throughout the change process” elicited a variety of responses. A minority of participants indicated definitively positive views of the effectiveness of communication. These interviewees described communications from MLA leadership as transparent, upfront, frequent, and felt that dialogue with membership about the transition process was successfully facilitated.

“They've been pretty upfront about it and why they needed to do it. That's good to hear.”

“What I think was part of the secret sauce … dialogue and listening, hearing an idea, [and] editing and modifying things.”

The majority of transcripts indicated mixed viewpoints in relation to the perception that there is almost always room for improvement with communication.

“Did [we] convey the need to change our communities? I would have to say yes we did … in retrospect, we can always find ways to improve the process of effectively conveying any information to membership and I would say most of those efforts would be focused around communication.”

News and updates about the Communities Transition were conveyed through various channels, including member emails, the *MLAConnect* newsletter, blog posts, section and SIG communication, and in-person at chapter and annual meetings. This variety may have contributed to why communications were considered ineffective by some participants. For example, interviewees mentioned that members may not have read the written communications. Additionally, there was referral to inconsistent or infrequent communication. Concerns were also expressed that the importance of the information was not highlighted as effectively or engagingly as it could have been.

“I think that if MLA had some kind of special communication, instead of only trying to communicate it in the large configuration of the membership, perhaps like a white paper or an overall electronic meeting.”

### Effective and ineffective communication channels

MLA's chosen communication channels appear to have influenced perceptions of the effectiveness of information transfer relating to the Communities Transition. The most effective communication channels from MLA leadership to members were considered to be the in-person Open Forums at annual meetings, followed by the *MLAConnect* newsletter, blog posts, the MLA website, and in-person meetings with sections and SIGs.

However, all the channels considered most effective by many of the interviewees were also considered ineffective by a number of participants, largely due to personal preferences in communication delivery mediums and time constraints for reading messages. Accordingly, expressed reasons for ineffective communication were that members may not read blog posts, that MLA's website is difficult to navigate and inaccessible due to the volume of information, that section and SIG leaders may not have consistently relayed pertinent information to members, and that the Open Forums did not provide enough opportunity for dialogue, particularly since not all members attend annual meetings.

“When we got the print MLA News I remember … reading it from cover to cover. I felt like I knew what was happening. Now, it's one of the many emails … In terms of reading every one every week, I can't personally say that I do that. So, I think I'm not that different than the majority of membership, where we're missing things.”

### Communication of need for change and the communities strategic goal

When asked to reflect on whether MLA successfully communicated the reason or need for the Communities Transition, a definitive “yes” was discerned from the majority of the transcripts, with others indicating a mixed response or inferring “no.” Relatedly, when asked if the change was tied to the strategic plan, a minority answered “yes,” with most indicating either “no” or that they did not know. Whether the transition was considered to be tied to MLA's strategic plan is likely linked to whether the need for change was believed to be successfully communicated.

“There was a goal created around the community … Did everyone see it, take it in, understand it at the time? I'm not positive.”

### Understanding the need for change

The interviewees further discussed why they thought the change had occurred. The most commonly perceived reasons were reducing silos to increase innovation through better collaboration, resource sharing, and communication; increasing members' engagement with the organization; decreasing membership cost; expanding the number of new faces within leadership and on committees; and diversifying programming and educational opportunities for members.

Additional reasons mentioned included more efficient resource sharing, improved organizational agility and sustainability, better representation of the increasingly diverse membership, and creating equity between sections and SIGs in terms of programming, funding, and board representation.

“People who belong to Sections are, I think, three times more likely to stay involved. The whole evolution is trying to make it easier for people to find that community that works for them, that appeals to them.”

“One of the key factors that helped me understand why this is really important is thinking about the Sections and SIGS—how one group had funds to do things and the others didn't. And that might [link to] how programming was done at MLA and how people might have felt they were connecting to MLA. So, one of the key things here was to make it more inclusive.”

Mixed and negative conceptualizations of the Communities Transition were influenced by lack of clarity in communication to section leaders and the wider membership. Some interviewees discussed a perception of MLA conducting a “money grab” from sections and a push for the dissolution of identity-based sections and SIGs.

“It was not well received by people who didn't see the need, why now, why don't we do it some other time. What's the problem with my section, I love it, and it's working fine. Why do you want to change it.”

“A lot of people got caught up in the financial part of it, you know, you're taking our money, we're no longer going to have any control of our money. And so a lot of the messaging got lost because that narrative was the loudest.”

**Theme two: Perceived leadership during the change process**

### Length and pace of the change process

The perceived length from board approval to implementation of the new structure was mixed, with individual answers varying from one to fifteen years, implying that some members were unaware of elements of the Transition. Documentation revealed that conversations about reorganization had been occurring for almost a decade prior to implementation, so it is unsurprising that long-standing MLA members thought the process took longer than the actual implementation period from 2017 to 2019.

Similarly, perceptions surrounding the pace of change were varied. Interviewees in higher leadership positions were more likely to feel that the pace of change was consistent up until its acceleration near the end, whereas general members and those in midlevel leadership positions tended to consider the Communities Transition rushed and unplanned.

“It felt very rushed. This is a major, major change and I personally believe it was just kind of thrown out there and then you know … MLA as a whole was running with it.”

“It's been sort of steady because I've been watching it develop over the years. If you weren't watching it, it probably for some people hit them out of nowhere and that's the thing we have to figure out how to reconcile.”

### Representativeness and power of the leadership team

Most interviewees felt that the change leadership teams were not representative of the general membership, with some stating that the mechanisms for how and why selections occurred were not clearly communicated.

“It's a challenge to know how this particular task force was composed.”

“Were they successful in building a group that represented the voices of MLA? That I'm not sure about, I think, for as many members as there are in MLA, maybe the group seemed kind of small.”

There was near-consensus that the Communities Transition leadership teams had enough power to successfully implement the change. Associated concerns included that SIG and section leaders were not empowered with enough information to lead discussions about the change process and that the teams may have had “too much” power.

“They were successful in that. I don't know how you could have done it with a better group. Given the fact that it had the potential to be so contentious, people really worked hard to listen to each other … So the answer to that is yes, the groups have enough power to lead the change within the structure of MLA.”

“I think more could have been done to empower those current chairs, presidents, co-chairs, incoming chairs to feel more comfortable leading these discussions, not just online, but especially at the business meeting at MLA.”

**Theme three: Perceived membership investment in change**

### Membership's positive or negative responses to change and associated reasons

Participants were asked for their perceptions of the MLA membership's response to the Communities Transition process. Most classified the general membership as having a “mixed” response, although some felt that the response by membership was “negative.” Commonly perceived reasons for the membership's negative or mixed response to change were loss of financial control of section treasuries, an associated loss of section autonomy, that the urgent need for change was not completely conveyed, that the benefits of change were not clearly communicated, that a lot of change was occurring at once, and ambiguity about the change. Interviewees felt MLA membership was either not informed or did not realize that the change process was intentionally iterative.

“I often felt confused as to exactly what stage we were in … It was not exactly an iterative process but something along those lines, which I had never heard before, and even understanding that helped things click a little bit and say, okay, well, this is why they don't have everything figured out yet.”

“One of our greatest challenges is we have a lot of people who don't deal well with ambiguity and saying that we're going to figure this out as we go along just makes a lot of people really nervous.”

### Feedback solicitation by leadership and mechanisms used

Interviewees largely agreed that membership feedback was consistently solicited, although some felt that input could have been invited sooner. According to the transcripts, feedback was gathered by MLA leadership through in-person and online forums, section and SIG leaders, and meetings with Section treasurers.

“I think really if they would have had a more public call for volunteers… they could say, hey, we appreciate your ideas and thoughts … why don't you come and help us figure this out, like bring those naysayers to the table”

“There was a lot of opportunity for input. The problem is that communication is two way. People who wanted to have a voice needed to step up and give that voice instead of somehow waiting for people to knock on their door and ask them.”

**Theme four: Instituting change and future recommendations**

### Clarifying and communicating policies and procedures to membership

Participants emphasized the importance of communicating deadlines, new procedures, and new policies widely, clearly, and often as the organization implements the new structure. Associated concerns were about unknown deadlines and criteria for new or repurposed organizational activities, such as scholarships. Additional concerns included management and logistics for caucuses and whether a policies and governance manual about the new MLA leadership structure would be circulated to all members.

“Suddenly we don't have direct access to the funds that we had, so we have to get approval. What do we do, and how do we do this with these other Caucuses that have self aligned on this Hub?”

### Undertaking evaluation and transparently communicating successes and challenges

Discussion occurred in multiple interviews about the need for short- and long-term evaluation of the success of the Communities Transition. Interviewees recommended evaluation focusing on whether the original objectives have been met, whether diversity in leadership has increased, the associated costs of the change process, and whether members feel more engaged with MLA. Interviewees emphasized the importance of including the voice of general membership through assessment to determine the success of the change from their perspective.

“I think that over the next year to three years there's also assessments [to be] put into place. So in the future, you'll see assessments and surveys to say like we're on the right pathway or we have to make some changes”

“You could have a survey to send out and get some anonymous feedback, because people will be honest.”

Frequent, transparent, and accessible communication was considered a leading factor for ensuring acceptance and long-term success of the Communities Transition. Accordingly, interviewees provided suggestions for an ongoing reciprocal communication strategy, including follow-up question and answer sessions, both online and at annual meetings; accessible avenues for caucuses to report successes and challenges; programming summarizing the strategic plan and explaining how the Communities Transition fits within it; and ongoing updates of successes in relation to the original objectives.

“We need to keep this conversation going and definitely MLA needs to make sure that they're getting feedback and input … you know, being very connected with these Domain Hubs and seeing what's going on and what are their concerns and issues”

“Provide a summary of where we are and what we've done and where we still have yet to go and to develop. So, it's almost like stepping back and having an interim evaluation … Share the overall process or the overall objectives, vision, and Strategic Plan with members of your organization.”

### Consistently promoting new structure to encourage engagement

Finally, the importance of consistent promotion was addressed by the participants. This was described as both consistency in promoting the successes and accomplishments of the Communities Transition, coupled with consistent messaging and terminology. Specific ideas included promoting standardized language to refer to the new structure, spotlighting caucuses and domain hubs on *MLAConnect* or through online introductory meetings to encourage people to join, and scheduling online informal caucus meetings to encourage engagement and networking.

“The solidifying really comes with the—[how] can we help new people engage in this way, [do we have] a structure that empowers them to make a difference in the organization, and does that generate a feeling of goodwill and buy-in to the process and to the organization?”

## DISCUSSION

This research uncovered perceived strengths of MLA's Community Transition in relation to the board's strategic vision, ability to build effective teams to lead the Transition, and removal of barriers to make change happen. A significant example of removing potential barriers was the membership's vote to change organizational bylaws to remove the language of sections and any codified roles, as well as language clarifying the purpose and stewardship of organizational dues [6]. These changes allowed for the formation of caucuses and domain hubs, the elimination of dues for sections, and the transfer of scholarship funds to MLA stewardship. Interviewees generally felt that the teams tasked with leading the Transition were competent and had the power they needed to effect change, though some felt they may have had too much power. Nearly every interviewee also touched on understanding that the need for change was related to removing barriers to participation and breaking down silos within the organization. This indicates that the MLA Board and Executive Committee were successful in communicating the vision and need for change to those leading the Communities Transition process.

In our opinion, MLA leadership considered and followed the Kotter model's steps to successfully implement the Communities Transition. However, the model as written does not do enough to encourage strategic communication. While communication is interwoven within each step of the model, it is not given its own point of focus, and strategies for success are not discussed. It became clear that communication was the main issue of concern and disappointment for interviewees. These issues may have been avoided if an analysis of membership's preferred communication modes had been undertaken and a strategy for timing, repetition, and mode of information delivery had been planned in advance for each stage of the Transition process. Continuity in messaging, particularly terminology, through a variety of formats and channels is essential to ensure that messages are not only being broadcast but are also received and understood by all members.

Study limitations include its specificity to MLA, along with the relatively small sample size of participants with a specific scope of responsibilities. These issues make it difficult to generalize the findings outside MLA or as representative of all MLA members. The methodology and study sample were heavily influenced by MLA's Rising Stars Program and association leadership, requiring approval before implementation. We believe that an original proposal, designed to survey all MLA membership regarding the Communities Transition, would have had more generalizable and less biased findings. However, the proposal was rejected by MLA executive and board leadership because they considered it too early in the change process to survey the full membership effectively. We hope that MLA executive and board leadership will see the value in a full membership survey and undertake this important step in evaluation soon.

Despite the limitations, we believe this case study adds valuable knowledge in the area of publicly available research on organizational change in library associations. The main goal of the project, to investigate the Communities Transition process and share findings to guide MLA in the future, was accomplished. By sharing our work, we hope it will lead to further evaluative research and public documentation of large organizational changes in libraries and library associations for the benefit of the profession.

### Recommendations

We have several recommendations for MLA regarding the improvement of communication strategies with membership. First, we suggest developing a communication plan that would account for several preferences and factors of importance to MLA members. To accomplish this, we suggest undertaking an analysis of MLA members' preferred modes of communication. For example, it is important to understand if there is a need for more visual and auditory communication strategies beyond basic text. Second, once preferred modes and styles of communication are determined, it would be beneficial to map all routes of outbound communication available to MLA leaders. The MLA website was often mentioned as unwieldy and disorganized for the type of information needed by general membership; an information mapping exercise could uncover ways to help improve website usability. Third, we recommend designating a small team or a point-person for each topic of communication to ensure continuity of messaging and to track which channels have been used and when. This could help address a common concern revealed in the interviews regarding whether representatives to communities were relaying information accurately and in a timely manner. A communication manager could ensure that terminology remains consistent, that all informational channels are being maximized, and that there are no perceived lulls in communication.

Reorganizing a professional association is a large undertaking with many intended and unintended consequences. A professional organization can be a source of stability, support, and comfort to its members. In the future, we advise MLA leadership to research and implement a trauma-informed approach to any large-scale changes that impact membership [7]. Utilizing this approach resists the retraumatization of members by assuming that all individuals have experienced traumatic events and currently have unresolved trauma that leads to fear and resistance toward uncertainty [7]. Incorporating the six principles of a trauma-informed approach with a well-planned communication strategy could encourage more positive feelings toward change.

We recommend that all MLA members remember that organizational leadership consists of volunteers from the general membership and to not only set aside time to read and reflect on communication from MLA leadership and staff but also to respond thoughtfully and respectfully to colleagues. We are all MLA, and being invested in bettering our organization requires us to trust each other and be both dedicated speakers and listeners in communicating.

Finally, we recommend that all library leaders and managers use an evidence-based approach when implementing organizational change and to document their work in academic publications. Popular organizational change literature is often written by those in the corporate sector and not based on research or reflection beyond personal experience. When conducting background research for literature on library-focused organizational change, we did not find any relevant articles as we believe documentation of this type tends to be kept internally and not publicly published (e.g., most documentation on MLANET requires a member login). Library and information associations are undoubtedly experiencing changes of a similar nature to MLA, and it is our hope that more public scholarship on organizational change processes will be undertaken by libraries and similar professional associations. The recent Association of College and Research Libraries (ACRL) publication *Fostering Change: A Team-Based Guide* will be particularly useful for others considering changes of this nature and scale within library organizations [8]. Because literature on library management and organizational change implementation is limited, we encourage looking to examples in nonprofit and education fields to build a base of evidence for action.

In conclusion, while the MLA Communities Transition is now implemented and the eight steps from Kotter's model were generally well executed to prepare for and implement the change, we believe that more

planning and focus on communication would have helped to reduce membership anxiety and negative reactions to the process. We hope the recommendations laid out in this discussion of our research, including recommendations from interviewees on conducting a general membership feedback survey, continued information campaigns, and documenting and promoting successes from the Transition, will be implemented by MLA leadership.

## Data Availability

Data associated with this article are available in Digital Scholarship@UNLV at <https://digitalscholarship.unlv.edu/libdatasets/3/>.

## SUPPLEMENTAL FILES

**Appendix A.** Participant interview request email

**Appendix B.** Participant interview questions

## References

[1] Medical Library Association: about: strategic plan [Internet]. Chicago, IL: The Association [cited 26 Jan 2021]. <https://www.mlanet.org/p/cm/ld/fid=153>.

[2] Medical Library Association: transforming MLA communities [Internet]. Chicago, IL: The Association [cited 26 Jan 2021].<https://www.mlanet.org/p/cm/ld/fid=1511>.

[3] Kotter JP. Leading change: why transformation efforts fail. Boston, MA: Harvard Business School Press; 2010.

[4] Electronic Code of Federal Regulations. Protection of Human Subjects, Definitions for Purposes of this Policy 2018. 45 CFR 46.102 Section I. [cited 06 Apr 2021]. Available from: https://www.ecfr.gov/on/2018-07-19/title-45/subtitle-A/subchapter-A/part-46#46.501.

[5] Braun V, Clarke V. Using thematic analysis in psychology. Qual Res Psychol. 2006;3(2):77–101.

[6] Brassil E. Proposed bylaws amendments update [Internet]. Chicago, IL: The Association [cited 26 Jan 2021]. <https://www.mlanet.org/p/bl/ar/blogaid=877>.

[7] Substance Abuse and Mental Health Services Administration. SAMHSA's concept of trauma and guidance for a trauma-informed approach. HHS Publication No. (SMA) 14–4884. Rockville, MD: Substance Abuse and Mental Health Services; 2014. Available from: https://ncsacw.samhsa.gov/userfiles/files/SAMHSATrauma.pdf.

[8] Marshall B, Brecher Cook D, Ippoliti C. Fostering change: a team-based guide. Chicago, IL: Association of College and Research Libraries; 2020. [cited 06 Apr 2021]. Available from: http://www.ala.org/acrl/sites/ala.org.acrl/files/content/publications/booksanddigitalresources/digital/FosteringChange.pdf.

