# Supplementary material for: We are MLA: a qualitative case study on the Medical Library Association's 2019 Communities Transition
Source: J Med Libr Assoc. 2022 Jan 1;110(1):34–42. doi: 10.5195/jmla.2022.1225 (PMC8830398; doi:10.5195/jmla.2022.1225)
Supplement: Supplementary file 1 — Appendix A. Participant interview request email [file jmla-110-1-34-s01.docx]

**Appendix A**

**Participant Interview Request Email**

Hello!

The MLA Rising Stars 2019-2020 cohort is writing to inquire if we might interview you as part of our Rising Stars project for this year. We are researching the community reorganization formally instituted in 2019, and you have been mentioned as a person who would be a good interview subject as regards the transition, whether because of your involvement with the transition or because of your role as MLA leadership at the time.

We aim to conduct interviews via Zoom between November 1 and December 20. The interviews should take 30 minutes to an hour, and will be recorded. The audio recordings will be kept until May 2020, and written transcripts may be kept up to a year. In presentations and writings, no names will be associated with any direct quotes chosen to be illustrative of a point, and the majority of information will be synthesized and presented through summary and thematic analysis. The questions are scripted and will all be asked of each interview subject. From our interviews, we hope to gain a better understanding of the community reorganization, and how we can take the lessons learned from the reorganization to future changes for MLA. We cannot do this project without the insight of key community members like you, and are very appreciative of your willingness to participate!

Please use this poll ([https://doodle.com/poll/mgdwif8hncnsnrbw](https://nam05.safelinks.protection.outlook.com/?url=https%3A%2F%2Furldefense.proofpoint.com%2Fv2%2Furl%3Fu%3Dhttps-3A__nam01.safelinks.protection.outlook.com_-3Furl-3Dhttps-253A-252F-252Fdoodle.com-252Fpoll-252Fmgdwif8hncnsnrbw-26data-3D02-257C01-257Ck.bartley-2540med.miami.edu-257Cbe87fb405e6a412751dc08d75bd7eddf-257C2a144b72f23942d48c0e6f0f17c48e33-257C0-257C0-257C637078855290182277-26sdata-3D0uhms-252Ffa5TGysqReJHr1M48x8R1yWWiuFCzbrl3xxS4-253D-26reserved-3D0%26d%3DDwMGaQ%26c%3DsJ6xIWYx-zLMB3EPkvcnVg%26r%3D3MIr5ee_a3TcGT6FV-s4m8aYEasnSC0qWccGH6Y_uf8%26m%3D1a_ndCBeeCsk7I8otwsCPJeHNyKGoE5lkUF5k6Y_BM4%26s%3Dl_OaSdDwnx5iAaAd9BJKPvRTgTgEpMvN7pV0SXzBKYo%26e%3D&data=02%7C01%7CElaina.J.Vitale%40dartmouth.edu%7Cf5e1bbe7d98e4742c6c908d75bde5c0f%7C995b093648d640e5a31ebf689ec9446f%7C0%7C0%7C637078882923454240&sdata=TuS8iJCMUsbAkcVl44FHnW3gTReqvFMpdtTsCvXDo24%3D&reserved=0)) to indicate your availability, focusing your availability on the month of November first. If we cannot find a time, need to reschedule, or schedule a second meeting, we will resend the link to this poll. We will reach out to you with a date and time for an interview as soon as possible.

Please let us know if you have questions or feedback, or wish to nominate another leader to be interviewed in your stead.

Best,

Kelsa Bartley

Kathryn Houk

Jane Morgan-Daniel

Elaina Vitale
