# Supplementary material for: We are MLA: a qualitative case study on the Medical Library Association's 2019 Communities Transition
Source: J Med Libr Assoc. 2022 Jan 1;110(1):34–42. doi: 10.5195/jmla.2022.1225 (PMC8830398; doi:10.5195/jmla.2022.1225)
Supplement: Supplementary file 2 — Appendix B. Participant interview questions [file jmla-110-1-34-s02.docx]

**Appendix B**

**Participant Interview Questions**

**A Study of the 2019 MLA Communities Reorganization and Recommended Guidelines for the MLA Board’s Role in Future Institutional Change**

**Transition Involvement**

- How have you been involved in this process?
- What has your role been?

**Create a sense of urgency**

- Did MLA convey the need to change its communities effectively to membership?

**Build a guiding coalition**

- Was MLA successful in building a group to lead the transition of its communities and communicating this to membership?
- Did the group have enough power to successfully lead the change?

**Form a strategic vision & initiatives**

- How were the vision and initiatives of the Communities Transition, as well as its tie-in to the strategic plan and goals of MLA, described and communicated to the general membership?
- Do you think this information was well communicated and well received; why or why not?

**Enlist a volunteer army**

- How and when did leadership bring MLA members into the process of the Communities Transition?
- Do you think the strategy(ies) employed was successful for both accomplishing the Transition smoothly and getting general membership invested in the change?

**Enable action by removing barriers**

- What kinds of unforeseen obstacles did you encounter or observe during the transition process and how were these dealt with?

**Generate short-term wins**

- What short-term successes have you seen so far and what successes do you hope to see in the next few years?

**Sustaining acceleration**

- Has the Communities Transition has moved forward at a consistent pace over the past 3 years?
- Has the Communities Transition process has lost some momentum or is it accelerating?
- What projects/ steps are needed to be done by membership to continue the acceleration of the process?

**Institute Change**

- What is MLA leadership’s plan for solidifying the new Communities structure after the initial transition from the previous structure?
